# Reimagining dementia screening: A stakeholder-informed perspective on artificial intelligence, digital biomarkers, and real-world implementation

**DOI:** 10.1177/25424823251395310

**Published:** 2025-11-10

**Authors:** Kevin Mekulu, Faisal Aqlan, Hui Yang

**Affiliations:** 1 Complex Systems Monitoring, Modeling and Control Laboratory, 8082 Pennsylvania State University, University Park, PA, USA; 2 5170 Center for Human Systems Engineering, University of Louisville, Louisville, KY, USA

**Keywords:** Alzheimer's disease, artificial intelligence, digital technology, delivery of health care

## Abstract

The approval of disease-modifying treatments for Alzheimer's disease demands a rethinking of cognitive screening. Drawing on over 180 stakeholder interviews from the NSF National I-Corps program, this perspective highlights barriers in current workflows, from time constraints in primary care to learning effects in long-term care, and presents innovation pathways centered on AI and digital biomarkers. Speech analysis, in particular, offers a scalable and cost-effective screening tool aligned with existing CPT codes. We outline implementation strategies and emphasize the urgent opportunity to align technological innovation with frontline clinical needs to ensure advances translate into meaningful patient and provider benefit.

## Introduction

The landscape of Alzheimer's disease (AD) treatment has fundamentally changed with the FDA approval of lecanemab and donanemab.^1,2^ These breakthrough treatments offer unprecedented opportunities for early intervention, yet our healthcare system's capacity to identify suitable candidates remains constrained by outdated screening approaches. Through extensive stakeholder engagement, we identify critical gaps and opportunities in current practice and outline pathways for innovation that could revolutionize cognitive screening across care settings. This urgency is amplified by evolving reimbursement models and the critical need to identify eligible patients before symptoms and individual cost burden become severe.

## Current challenges and stakeholder perspectives

Our engagement with 180 healthcare professionals revealed systemic challenges across the care continuum. Primary care providers (PCPs), facing significant time constraints, struggle with traditional tools that can consume up to half of a patient visit. Moreover, only about 21% of PCPs report high confidence in recognizing neurocognitive disorders, and similarly, only 20% express high confidence in interpreting cognitive test results,^3^ underscoring the need for more objective assessment methods.

These stakeholder insights were collected through semi-structured interviews with clinicians, senior living administrators, and caregivers conducted as part of the NSF I-Corps program. Interview feedback was thematically synthesized to identify recurring implementation barriers and innovation priorities across care settings.

Long-term care facilities face their own critical challenges with current screening approaches. Traditional assessment tools suffer from significant learning effects, where residents become familiar with the tests over repeated administrations (every quarter), compromising the validity of longitudinal monitoring. This challenge is particularly acute in memory care units and skilled nursing facilities, where continuous monitoring of disease progression is essential. These issues are further compounded by widespread staffing shortages and high turnover rates, which affect the consistency and quality of assessments.

Cost barriers create additional complications across settings. While PET scans offer diagnostic precision, their high cost limits accessibility, particularly during early stages when treatments prove most effective. As one neurologist emphasized, “The best treatments work when patients are in a very mild state, but not everyone can get PET scans due to pricing issues.” This challenge is compounded by the fact that approximately two-thirds of Alzheimer's patients experience “denial of illness,” making early detection and intervention even more crucial.^4^

## Innovation opportunities

### Artificial intelligence and digital biomarkers

The convergence of AI capabilities with healthcare needs presents several promising opportunities. Healthcare providers emphasized the critical need for tools that can differentiate between various types of dementia (frontotemporal, Huntington's, vascular, AD, etc.). While current screening tools like the AD8 demonstrate sensitivity and specificity around 80% for general cognitive impairment, neurologists in our study indicated that an AUC of 70% would be clinically acceptable for an AI-powered differential diagnosis tool.

AI-powered speech analysis shows early promise as a scalable approach, though current findings remain preliminary and should be interpreted as proof of concept.^5,6^ References 5 and 6 are currently available as preprints on medRxiv and should be interpreted as preliminary findings pending peer review. In independent and assisted living settings, these tools could detect subtle cognitive changes through natural conversations. In memory care units and skilled nursing facilities, they offer a way to track disease progression while avoiding the learning effects of traditional tests. For primary care settings, where rapid assessment is crucial, speech analysis could provide quick (under 5 min), sensitive, and specific screening results while maintaining natural patient interaction. This approach is particularly relevant now that cognitive screening is supported by CPT codes (#99483), offering a practical path to implementation in primary care workflows. Beyond speech, stakeholders expressed interest in the future integration of eye-tracking, motor coordination, and neurophysiological signals (e.g., dry-electrode EEG) to support differential diagnosis and longitudinal monitoring.

### Care delivery innovation

Beyond screening tools, stakeholders identified opportunities for broader care improvement. Generative AI (GenAI) through large language models (LLMs) could help address staffing challenges by streamlining care plan development while maintaining personalization. It's important to note that GenAI is exploratory and not meant to replace clinician judgement.

In skilled nursing facilities and memory care units, administrators emphasized the need for technologies that enhance residents’ comfort and joy while addressing practical concerns like fall prevention, which was identified as the greatest risk in these settings. As one administrator noted, “We need solutions that make residents comfortable and bring joy to their lives.”

**Figure 1.**
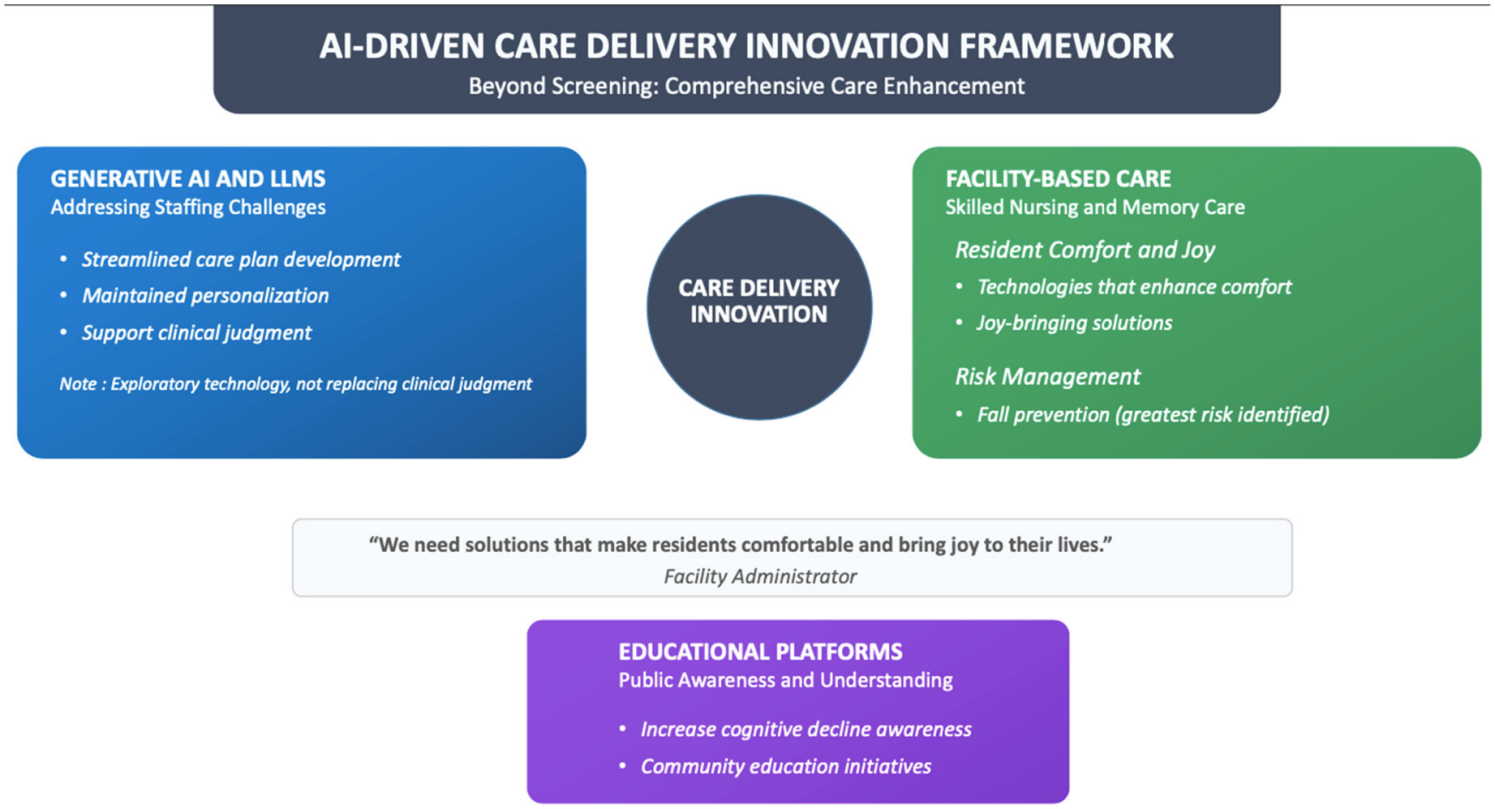
Framework for AI-driven care delivery innovation beyond screening, integrating generative AI, facility-centered technologies, and public education. This tripartite model addresses critical gaps in dementia care—streamlining clinical workflows, enhancing resident experience, and elevating community understanding. Anchored by stakeholder feedback, the framework highlights scalable domains for improving care personalization, mitigating fall risks, and fostering joy in institutional settings.

Educational platforms represent another critical opportunity, with stakeholders highlighting the importance of increasing public awareness and understanding of cognitive decline Figure 1.^7^

The three axes correspond to generative AI for workflow automation, facility-centered technologies for safety and comfort, and public education for awareness, together forming a balanced model of innovation.

## Implementation consideration

Acceptance and successful implementation of these technologies require careful attention to several critical factors:

### EHR integration and workflow

Solutions must integrate seamlessly with existing Electronic Health Record (EHR) systems and clinical workflows. While EHR integration challenges are well-documented in healthcare innovation,^8^ our stakeholders consistently emphasized that new screening tools must avoid creating additional documentation burden and instead work within existing EHR infrastructure. This integration is particularly crucial in time-constrained settings like primary care, where efficiency and accessibility of information directly impact adoption rates.

### Setting-specific design

Tools must adapt to the unique constraints and objectives of different care environments. Independent and assisted living facilities require solutions focused on early detection and subtle changes, while memory care and skilled nursing facilities need robust disease progression monitoring capabilities. Primary care settings demand rapid, efficient tools that can seamlessly integrate into brief patient visits while maintaining high sensitivity and specificity.

### Staff engagement and training

While new technologies can reduce overall burden, they require thoughtful implementation and training programs to ensure effective adoption, interpretation, and responsive and appropriate adjustments to case management. This is particularly important given the widespread staffing challenges across care settings. Implementation strategies must account for varying levels of technological expertise and high staff turnover rates, emphasizing intuitive interfaces and streamlined training processes.

### Equity and generalizability

AI-based screening tools must be validated across diverse racial, ethnic, linguistic, and socioeconomic populations to ensure equitable performance and avoid bias. Model development and deployment should incorporate representative datasets and transparent reporting of limitations.

## Path forward

**Figure 2.**
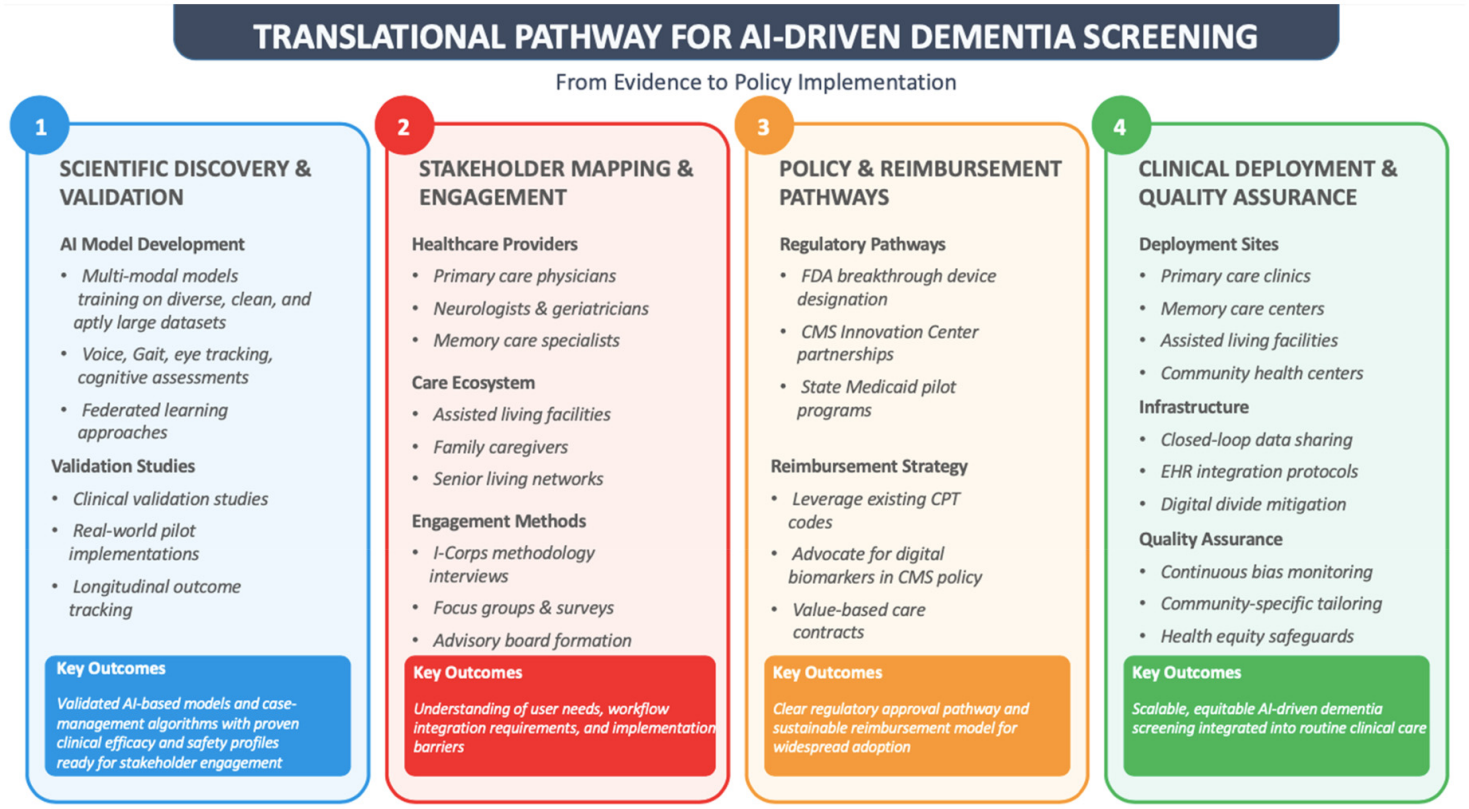
Translational pathway for AI-driven dementia screening, highlighting a four-stage pipeline from foundational scientific discovery to real-world policy implementation. Each layer reflects an essential component: algorithm validation, stakeholder alignment, regulatory strategy, and clinical integration. Together, this schema defines a realistic trajectory for scaling digital biomarkers within diverse care settings, while anticipating reimbursement, infrastructure, and equity-related challenges.

The transformation of dementia screening requires coordinated effort across multiple dimensions, from scientific validation to policy integration and real-world implementation. To bridge this gap, we propose a translational pathway that synthesizes foundational discovery, stakeholder feedback, reimbursement planning, and deployment strategy. This framework illustrated in Figure 2, offers a structured, actionable model for advancing AI-powered dementia screening into scalable clinical use.

Each column represents a translational layer: Scientific validation, stakeholder alignment, policy and regulatory preparation, and clinical deployment, defining a sequential path from research to real-world practice.

## Technology development

Development efforts should focus on validated digital biomarkers and integrated assessment approaches that can provide objective, continuous assessment of cognitive function. Particular attention should be paid to ensuring high sensitivity and specificity while maintaining practical usability across care settings.

## Clinical validation

Clear evidence must be established for the sensitivity, specificity, and clinical utility of new screening approaches. This includes validation across different patient populations and care settings, with careful attention to factors that could affect performance and reliability. Multi-center studies will be needed to validate these tools across diverse healthcare settings with particular emphasis on establishing appropriate clinical thresholds and understanding potential confounding factors.

## Implementation research

Best practices for technology adoption must be developed and refined across different care settings, taking into account workflow integration, staff training needs, and setting-specific requirements.

## Conclusions

The imperative for better dementia screening tools has never been stronger. Our stakeholder analysis reveals both significant challenges in current practice and promising opportunities for innovation. By leveraging emerging technologies like AI-powered speech analysis and automated personalized care planning, we can create more effective, efficient, and accessible screening approaches. Success will require careful attention to implementation considerations and close collaboration with frontline healthcare providers. With treatments now available, the convergence of technology, clinical urgency, and healthcare policy creates a once-in-a-generation opportunity to reimagine dementia screening. To better serve patients and optimally support clinical care, dementia screening must evolve into a dynamic, equitable, and implementation-ready process.
